# Stimulated hepatic stellate cell promotes progression of hepatocellular carcinoma due to protein kinase R activation

**DOI:** 10.1371/journal.pone.0212589

**Published:** 2019-02-22

**Authors:** Yusuke Imai, Osamu Yoshida, Takao Watanabe, Atsushi Yukimoto, Yohei Koizumi, Yoshio Ikeda, Yoshio Tokumoto, Masashi Hirooka, Masanori Abe, Yoichi Hiasa

**Affiliations:** Department of Gastroenterology and Metabology, Ehime University Graduate School of Medicine, Shitsukawa, Toon, Ehime, Japan; University of Navarra School of Medicine and Center for Applied Medical Research (CIMA), SPAIN

## Abstract

Hepatic stellate cells (HSCs) were reported to promote the progression of hepatocellular carcinoma (HCC), however its mechanism is uncertain. We previously reported that protein kinase R (PKR) in hepatocytes regulated HCC proliferation. In this study, we focused on the role of PKR in HSCs, and clarified the mechanism of its association with HCC progression. We confirmed the activation of PKR in a human HSC cell line (LX-2 cell). IL-1β is produced from HSCs stimulated by lipopolysaccharide (LPS) or palmitic acid which are likely activators of PKR in non-alcoholic steatohepatitis (NASH). Production was assessed by real-time PCR and ELISA. C16 and small interfering RNA (siRNA) were used to inhibit PKR in HSCs. The HCC cell line (HepG2 cell) was cultured with HSC conditioning medium to assess HCC progression, which was evaluated by proliferation and scratch assays. Expression of PKR was increased and activated in stimulated HSCs, and IL-1β production was also increased molecular. Key molecules of the mitogen-activated protein kinase pathway were also upregulated and activated by LPS. Otherwise, PKR inhibition by C16 and PKR siRNA decreased IL-1β production. HCC progression was promoted by HSC-stimulated conditioning medium although it was reduced by the conditioning medium from PKR-inhibited HSCs. Moreover, palmitic acid also upregulated IL-1β expression in HSCs, and conditioning medium from palmitic acid-stimulated HSCs promoted HCC proliferation. Stimulated HSCs by activators of PKR in NASH could play a role in promoting HCC progression through the production of IL-1β, via a mechanism that seems to be dependent on PKR activation.

## Introduction

The incidence and mortality of hepatocellular carcinoma (HCC) is one of the highest among malignant tumors worldwide [1]. The incidence of HCC caused by hepatitis virus has decreased due to advances in antiviral therapy, although HCC caused by nonalcoholic steatohepatitis (NASH) has been increasing [2, 3]. Although the prognosis of early to moderate stage HCC has improved due to the development of treatment strategies [4], advanced stages of HCC still carry a poor prognosis [5]. Progression of HCC is affected by the hepatic microenvironment, which consists of various non-parenchymal and parenchymal cells and soluble factors [6, 7]. Manipulation of the microenvironment may be a therapeutic target for inhibiting HCC development.

Hepatic stellate cells (HSCs) form a major component of the non-parenchymal cells in the liver and are involved in forming the microenvironment. HSCs are located in the space of Disse in the liver, and store vitamin A intracellularly during the quiescent phase [8, 9]. Once HSCs are activated by various stimuli, including cytokines, pathogen associated molecular patterns (PAMPs) and damage associated ones (DAMPs), they begin secreting extracellular matrix and promote liver fibrosis [10–13]. In case of NASH, lipopolysaccharides (LPS) and palmitic acid flowing into the portal vein from the intestinal tract activate HSCs and promote collagen production [14–17]. Thus, HSCs play a central role in the development of liver cirrhosis. Recent papers have shown that HSCs contribute to the progression of HCC by secreting various inflammatory cytokines, including IL-1β [18–20]. However, the mechanisms by which HSCs secrete inflammatory cytokines and influence HCC progression are not well understood.

PKR is a double-stranded, RNA-dependent protein kinase that is induced by interferon. It is a key executor of antiviral responses, although recent studies have revealed its important role in malignant diseases. We previously reported that PKR in hepatocytes regulate not only innate immunity as HCV elimination, but also cell proliferation as HCC development [21–24]. In macrophages, LPS-induced cell activation is mediated by PKR [25]. Further, PKR in macrophages regulates production of inflammatory cytokines through mitogen-activated protein kinase (MAPK) pathways [25, 26]. Thus, PKR is regarded as a key regulator of inflammatory cytokine production.

Given these facts, we hypothesized that PKR in HSCs might regulate inflammatory cytokine production, and that the cytokines released by HSCs might alter the microenvironment and accelerate HCC progression. However, both the expression and role of PKR in HSCs are poorly understood. The aim of this study was to investigate the expression of PKR in HSCs and to clarify the role of PKR in HSCs in relation to HCC progression.

## Materials and methods

### Cell lines

The human HSC cell line LX-2 was purchased from Merck (Darmstadt, Germany). LX-2 was cultured with Dulbecco’s modified Eagle medium without glutamine, (DMEM; Thermo Fisher Scientific, Waltham, MA, USA) supplemented with 2% fetal bovine serum (FBS; Merck), 2mM L-Glutamine (Thermo Fisher Scientific) and 1% penicillin and streptomycin. Cells of the human HCC cell line HepG2 (Japanese Collection of Research Bioresources, Osaka, Japan) were cultured with high glucose DMEM (Thermo Fisher Scientific) supplemented with 10% FBS and 1% penicillin and streptomycin. Cells were maintained at 37°C in a humidified atmosphere of 5% CO_2_ and 95% air, and the culture medium was changed three times per week.

### Preparation of palmitic acid

0.0256 μg of palmitic acid was added to 1000 μL of 99% ethanol and heat at 50°C for 3 min. 1.0g of BSA was dissolved in 9ml water and 40 μL of 1N NaOH, heated at 50°C for 3 min. 100 μL of palmitic acid solution were added in 900μL of BSA solution.

### HSC activation

Lipopolysaccharides (LPS; Merck) at a concentration of 100 ng/mL or tumor growth factor-β (TGF-β; Merck) at a concentration of 3.5 ng/mL were added to the DMEM and the cells were cultured for 24 hours for LX-2 cell (1.5×10^5^ /well) activation. Separately, palmitic acid (200 nM) (Merck) was added to the DMEM and the cells were cultured for six hours for LX-2 cell activation. For inhibiting activation of LX-2 cells, 200 nM C16, a PKR inhibitor (Merck) or 100 μM JNK inhibitor Ⅱ (Merck) or 40 μM U0216, a MEK inhibitor (Merck) was supplied one hour before addition of LPS or palmitic acid.

### Collection of conditioning medium (CM) from HSCs

For collection of CM, LX2 was seeded onto a 30 mm dish. The following day, cells were incubated with serum free DMEM for 24 hours. For collection of CM from activated HSCs, LPS or palmitic acid was added to serum free DMEM for 24 hours (LPS) or six hours (PA). For PKR blocking, C16 was added one hour before supplementation of LPS or palmitic acid.

### RNA interference

PKR-targeting siRNA (GAG AAU UUC CAG AAG GUG A) was designed using a PKR sequence template (accession number NM002759), and was then synthesized by Thermo Fisher Scientific. Control siRNA was purchased from GE Healthcare (Tokyo, Japan). LX-2 cells at 50% confluence in 6 well plates were transfected with 50 pM siRNA using RNAiMAX (Merck).

### Real-time polymerase chain reaction (RT-PCR) testing

Total RNA was extracted from LX-2 cells using RNeasy Plus Mini Kit (QIAGEN, Venlo, Netherlands) according to the manufacturer’s instructions. Total RNA was reverse transcribed using the High Capacity cDNA Reverse Transcription Kit (Thermo Fisher Scientific) according to the manufacturer’s instructions. RT-PCR testing was performed using the LightCycler system (Roche Diagnostics, Mannheim, Germany) and 5 μL of SYBR Green mixture (Roche), 2 μL of purified cDNA, and 1 μL of forward and reverse primer were used for PCR amplification. The primer sequences used were GAPDH (forward primer: 5’-agccacatcgctcagacac-3’, reverse primer: 5’-gcccaatacgaccaaatcc-3’), IL-1β (forward primer: 5’-GCCAGTGAAATGATGGCTTAT-3’, reverse primer: 5’-ggtcctggaaggagcactt -3’), and PKR (forward primer: 5’-AGCACACTCGCTTCTGAATC-3’, reverse primer: 5’-CTGGTCTCAGGATCATAATC-3’). Relative mRNA expression levels of target host genes were defined by dividing their values by the amount of GAPDH mRNA, which were then evaluated by statistical analysis.

### Enzyme-linked Immunosorbent Assay for IL-1β

Concentrations of IL-1β in LX-2 cell lysates were measured using an Enzyme-linked Immunosorbent Assay (ELISA) kit (R&D Systems, Minneapolis, MN, USA) following the manufacturer’s instructions. The lower limit of detection for IL-1β was 1 pg/ml.

### Western blotting

For Western blotting, 20 μg proteins were applied to lanes of 10% or 4% to 12% Bis-Tris Gels, and then transferred onto Immobilon-P membranes (Millipore, Bedford, MA, USA). The membranes were incubated with the relevant antibodies: anti-PKR (product number: 3210), anti-EIF2α (5324), anti-phospho EIF2α (3398), anti-c-Jun (9165), anti-phospho c-Jun (3270), anti-c-Fos (2250), anti-phospho c-Fos (5348), anti-JNK (9252), anti-phospho JNK (4668), anti-ERK1/2 (4659), anti-phospho ERK1/2 (4370) (Cell Signaling, Danvers, MA, USA), and phospho PKR (44-668G) (Thermo Fisher Scientific), overnight at 4°C. Appropriate species-specific conjugated secondary antibody kits were commercially obtained (GE Healthcare, Charles Coffin, NY, USA). Signals were detected using the ECL Prime Kit (GE Healthcare) with an ImageQuant LAS 4000 system (GE Healthcare).

### Proliferation assay/Cytotoxicity assay

LX-2 cells were seeded onto 96 well plates (4000 cells/well) for cytotoxicity assay and cultured with DMEM supplemented with 2% FBS for twenty-four hours. Then, LX-2 cells were cultured with C16 for twenty-four hours or transfected with siRNA and cultured for seventy-two hours and cell viability was evaluated. HepG2 cells were seeded onto 96 well plates (4000 cells/well) for proliferation assay and cultured with DMEM supplemented with 10% FBS. Twenty-four hours later, the supernatant was replaced with 100 μL CM (non-stimulated, LPS-stimulated, palmitic acid-stimulated, pretreated with C16 before stimulation) for twenty-four hours later and cell proliferation was assessed. Cell viability and proliferation was spectroscopically assessed using the Cell Counting Kit 8 (CCK8; FUJIFILM Wako Pure Chemical Corporation, Osaka, Japan) and light absorbance (optical density (OD)) of each well at 450nm was noted.

### Scratch assay

HepG2 cells were seeded on a six well plate (3×10^5^ cells/well) and cultured with DMEM supplemented with 10% FBS. Twenty-four hours later, the cell layers were scratched with the tip of a pipette and the supernatant was replaced with 2 mL CM (non-stimulated, LPS-stimulated, palmitic acid-stimulated, pretreated with C16 before stimulation). The width of the wound was measured using a photomicroscope, a compact, inverted microscope Axio Vert A1 (Leica Microsystems, Wetzlar, Germany) with an objective lens (magnification 2.5x and numerical aperture 0.25) and a microscope camera Axio Cam MR3 (Leica).

The percentage wound area that was filled with proliferated HepG2 cells for 24 hours was calculated as follows: {(mean wound breadth–mean remaining breadth)/ mean wound breadth} × 100 (%). Mean breadth was measured by acquisition software (Axio Vision 4.8).

### Invasion assay

Twenty-four well, 8 μm pore size Matrigel invasion chambers (Corning Inc., NY, USA) were used for invasion assay. HepG2 cells were seeded into the upper chamber (1×10^5^ cells/well) and cultured with serum free DMEM. CM (non-stimulated, LPS-stimulated, pretreated with C16 before stimulation) was added into the lower compartment. Twenty-four hours later, the non-invading cells remaining in the upper chamber were removed with a cotton swab. The invading cells were fixed with methanol and stained with 0.05% toluidine blue (FUJIFILM Wako Pure Chemical Corporation, Osaka, Japan). Invading cells were counted at 20× magnification in three microscopic fields for each membrane.

### Statistical analysis

All statistical analyses were performed using JMP 11 software (SAS Institute, Tokyo, Japan). Data are expressed as the mean and standard error (SE). Statistical differences were determined using Student’s t-test.

## Results

**Fig 1:**
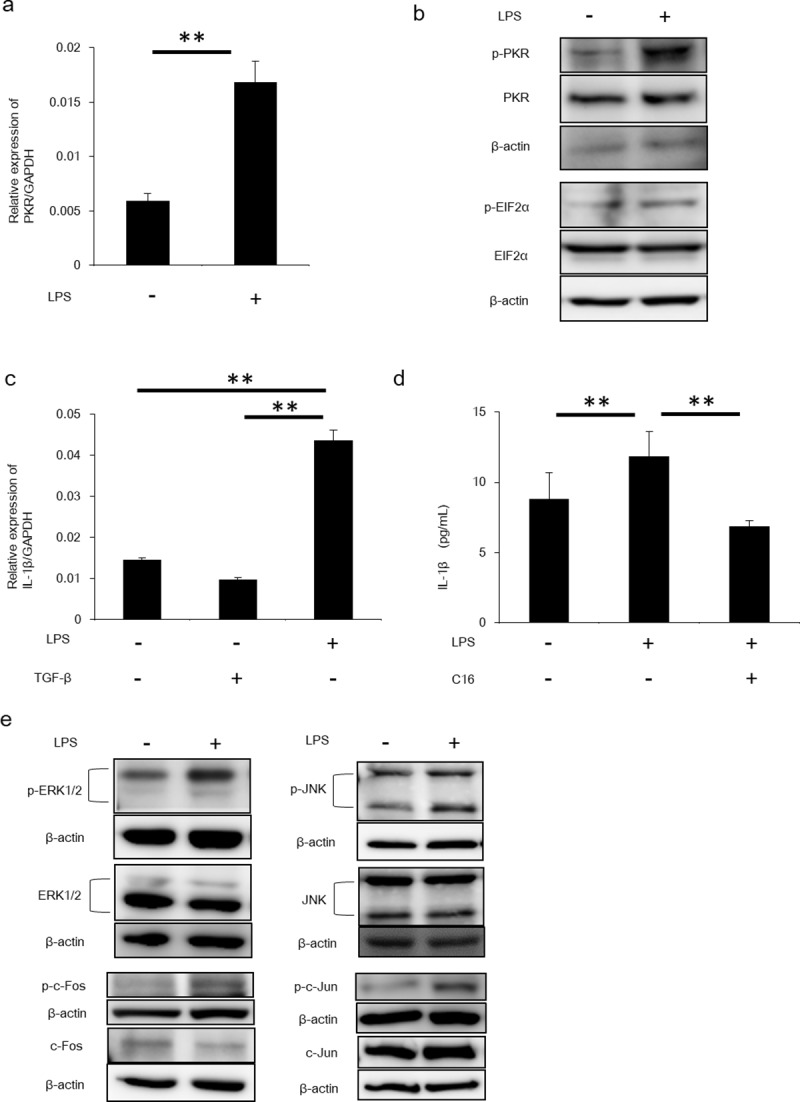
LPS promoted IL-1β production from HSCs via PKR signalling. (A) PKR mRNA in HSCs stimulated by LPS was measured by RT-PCR. Mean ± SEM of four replicates. **P<0.05. (B) Protein expression of PKR and EIF2α and each phosphorylated form was determined by Western blotting. (C) LX-2 cells were stimulated by LPS or TGF-β, following which mRNA levels of IL-1β were quantified by RT-PCR. Mean ± SEM of four replicates. **P<0.05. (D) The amount of IL-1β in LX-2 cells stimulated by LPS and inhibition by C16 was measured by ELISA. Mean ± SEM of three replicates. **P<0.05. (E) Upregulation of PKR mRNA by LPS stimulation was measured by RT-PCR. Mean ± SEM of four replicates. **P<0.05. Protein expression of JNK, ERK1/2, c-Jun and c-Fos and each phosphorylated form were determined by Western blotting.

PKR expression and IL-1β secretion by HSCs was upregulated by LPS stimulation First, we evaluated and ensured the expression of PKR in HSCs (Fig 1A and 1B). The amount of phosphorylated PKR in HSCs was increased by LPS stimulation (Fig 1B). Further, EIF2α, which was phosphorylated by phosphorylated PKR, was also induced by LPS stimulation (Fig 1B).

LPS is known to induce hepatic injury. Further, serum levels of LPS correlate with the severity of NASH and ASH [14]. Recent studies indicated that HSCs were stimulated by LPS and released various inflammatory cytokines [20]. Hence, we evaluated IL-1β expression in HSCs stimulated by LPS. IL-1β expression and secretion from LX-2 cells were increased after LPS stimulation (Fig 1C and 1D). However, TGF-βdid not increase IL-1β expression in HSCs as previous study (Fig 1C) [20]. These results demonstrate that LPS stimulation upregulated PKR expression in HSCs and promoted IL-1β production by HSCs.

### HSC activation by LPS was mediated by the PKR/MAPK pathway

The MAPK pathway is a key pathway in macrophage activation downstream of PKR [25]. We measured several MAPK pathway molecules in LPS-stimulated HSCs. Phosphorylated JNK, phosphorylated ERK, phosphorylated c-Jun and phosphorylated c-Fos were increased after LPS stimulation (Fig 1E). These results demonstrate that the PKR/MAPK pathway in HSCs was upregulated by LPS stimulation and was involved in induction of IL-1β secretion.

### PKR/MAPK pathway inhibition suppressed IL-1β production

**Fig 2:**
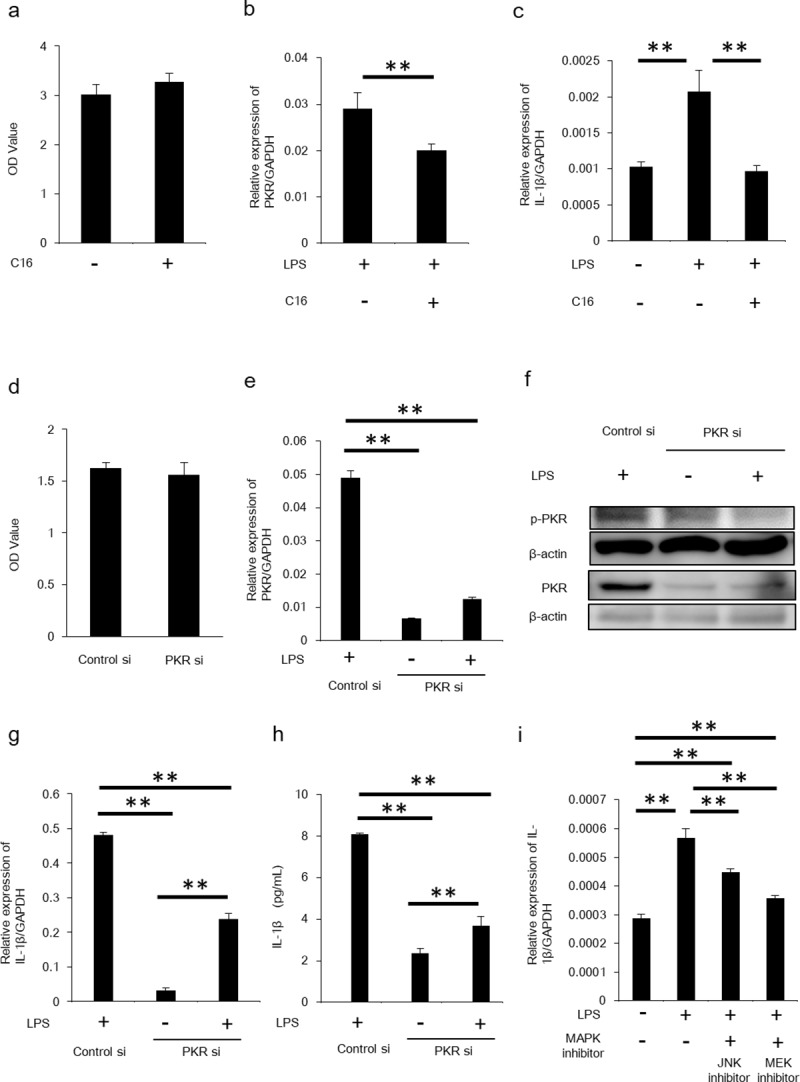
IL-1β was downregulated by inhibition of PKR. (A) Cytotoxicity assay was performed for assessing cell viability of C16-treated LX-2 cells. Mean ± SEM of eight replicates. **P<0.05. (B) Following LX-2 cells stimulated by LPS and inhibition by C16, PKR mRNA was quantified by RT-PCR. Mean ± SEM of four replicates. **P<0.05. (C) IL-1β mRNA in LX-2 cell stimulation by LPS and inhibition by C16 was measured by RT-PCR. Mean ± SEM of four replicates. **P<0.05. (D) Cytotoxicity assay was performed for assessing cell viability of siRNA-treated LX-2 cells. Mean ± SEM of eight replicates. **P<0.05. (E) LX-2 cells were transfected with PKR siRNA or control siRNA and stimulated by LPS. PKR mRNA was measured by RT-PCR. Mean ± SEM of three replicates. **P<0.05. (F) Protein expression of phosphorylated forms of PKR were determined by Western blotting. (G) IL-1β mRNA in LX-2 transfected with PKR siRNA was measured by RT-PCR. Mean ± SEM of three replicates. **P<0.05. (H) The amount of IL-1β in LX-2 transfected with PKR siRNA was measured by ELISA. Mean ± SEM of three replicates. **P<0.05. (I) Following LX-2 cell stimulation by LPS and inhibition by JNK or MEK inhibitor, IL-1β mRNA was quantified by RT-PCR. Upregulation of PKR mRNA was inhibited by MAPK inhibitors. Mean ± SEM of four replicates. **P<0.05.

In order to ensure the involvement of PKR/MAPK pathway in IL-1β secretion by HSC, we blocked PKR in HSCs by the PKR inhibitor, C16, and siRNA. C16 did not the cell viability of LX-2, as confirmed by cytotoxicity assay (Fig 2A). IL-1β expression in LX-2 cells was reduced by C16 (Fig 2B and 2C). IL-1β secretion from LX-2 cells was also reduced by the PKR inhibitor (Fig 1D). A similar phenomenon was observed by knocking down PKR by PKR siRNA. SiRNA did not affect cell viability at the concentrations in our experiments, as confirmed by cytotoxicity assay (Fig 2D). Inhibition of PKR expression in LX-2 cells by PKR siRNA downregulated mRNA levels of IL-1β and reduced IL-1β production by LX-2 (Fig 2E, 2F, 2G and 2H). MAPK inhibitors (JNK inhibitor and MEK inhibitor) also suppressed expression of IL-1β in LX-2 cells stimulated by LPS (Fig 2I). These results indicated that PKR/MAPK pathway is a key molecule in IL-1β production by HSCs.

### PKR in activated HSCs regulated HCC progression

**Fig 3:**
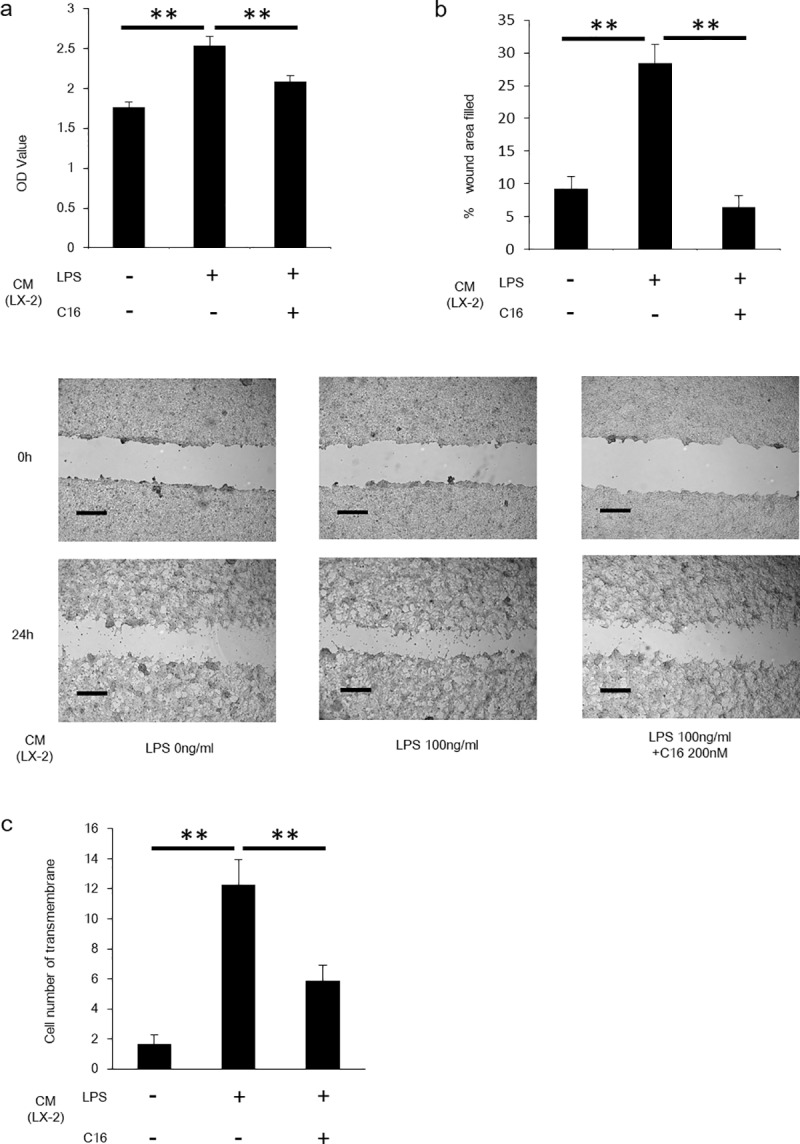
LPS-stimulated HSCs increased proliferation and invasiveness of HepG2 cells. (A) Proliferation assay was performed for assessing proliferation of HepG2 cells incubated by conditioning medium from LPS-stimulated or C16-treated LX-2 cells. Mean ± SEM of eight replicates. **P<0.05. (B) Scratch assay was performed for assessing proliferation of HepG2 cells incubated by conditioning medium from LPS-stimulated or C16-treated LX-2 cells. Scale bars represent 500 μm. Mean ± SEM of four replicates. **P<0.05. (C) Invasion assay was performed for assessing invasiveness of HepG2 was performed for assessing proliferation of HepG2 cells incubated by conditioning medium from LPS-stimulated or C16-treated LX-2 cells. Mean ± SEM of four replicates. **P<0.05.

HSCs are involved in carcinogenesis and the progression of HCC [18]. In this study, we investigated whether PKR in HSCs contributes to HCC development. HepG2 cells were cultured with CM from LPS-stimulated LX-2 cells with/without PKR inhibitor pretreatment. CM from LPS-stimulated LX-2 cells induced more HepG2 cell proliferation compared with CM from LX-2 without LPS stimulation. Further, Pretreatment with the inhibitor in HSC stimulation significantly prevented the HCC progression. The findings were confirmed by proliferation assay and scratch assay (Fig 3A and 3B). To ensure the promotion of HCC invasion by HSCs, invasion assay was performed under the same experimental conditions. Invasion of HepG2 cells were more frequent when HepG2 cells were cultured with CM from LPS-stimulated LX-2 cells. Further, HepG2 invasion was suppressed by HSCs pretreated with C16 (Fig 3C). These results demonstrated that progression and invasiveness of HCC was mediated by HSCs and regulated by PKR in HSCs.

**Fig 4:**
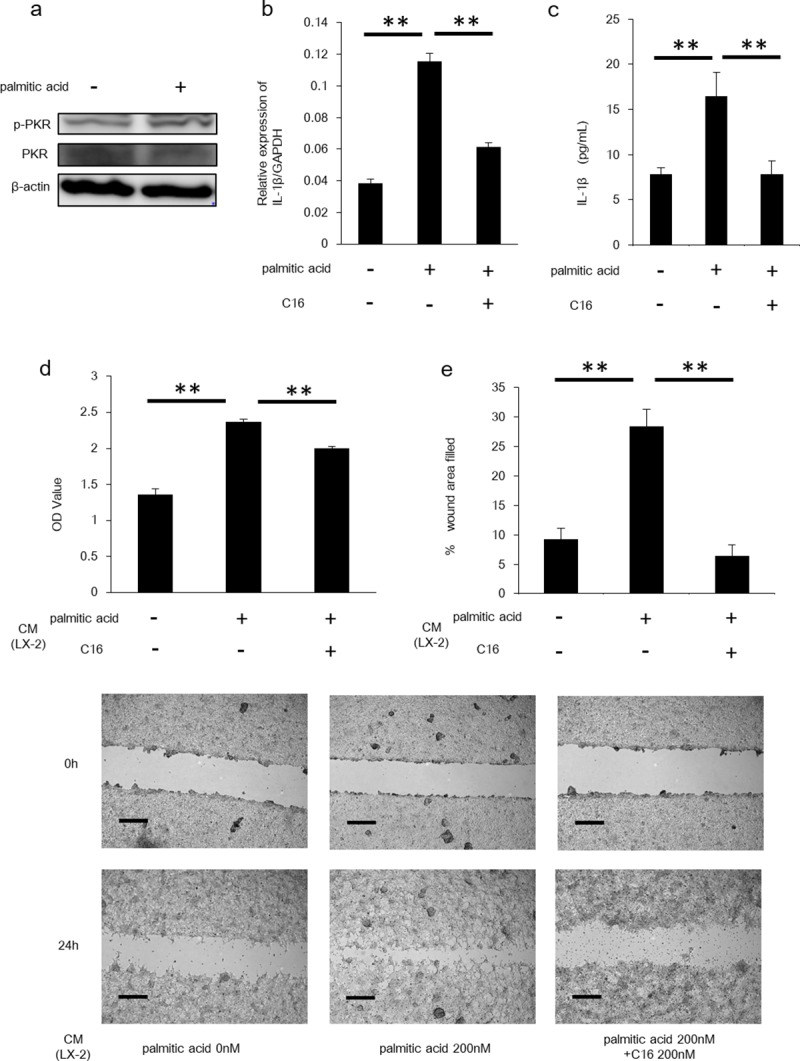
Palmitic acid-stimulated HSCs demonstrated increased IL-1β expression and enhanced the proliferation of HepG2 cells. (A) Protein expression of PKR and its phosphorylated form was determined by Western blotting. (B) Following stimulation of the LX-2 cells by palmitic acid, IL-1β mRNA levels were quantified by RT-PCR. Mean ± SEM of four replicates. **P<0.05. (C) The amount of IL-1β in LX-2 cells was measured by ELISA. Mean ± SEM of three replicates. **P<0.05. (D) Proliferation assay was performed for assessing proliferation of HepG2 cells incubated by conditioning medium from palmitic acid-stimulated or C16-treated LX-2 cells. Mean ± SEM of six replicates. **P<0.05. (E) Scratch assay was performed for assessing proliferation of HepG2 cells incubated by conditioning medium from palmitic acid-stimulated or C16-treated LX-2 cells. Scale bars represent 500 μm. Mean ± SEM of four replicates. **P<0.05.

Palmitic acid enhanced PKR expression in HSCs and promoted HCC proliferation PA binds to TLR-4 and exacerbates the pathogenesis of NASH by accelerating hepatocyte injury and inflammation [15, 16]. We hypothesized that PA might regulate IL-1β production from HSCs via PKR induction, and that IL-1β from HSCs might promote HCC development. The amount of phosphorylated PKR in HSCs was increased by PA stimulation (Fig 4A). And as expected, PA enhanced the production of IL-1β in LX-2 cells (Fig 4B and 4C). The enhancement was diminished by inhibition of PKR (Fig 4B and 4C). Furthermore, HCC proliferation was enhanced by CM from PA-stimulated HSCs and the proliferation was suppressed by PKR inhibition (Fig 4D and 4E). These results indicated that PA may be involved in the progression of HCC and that this progression is mediated by PKR in HSCs.

## Discussion

HSCs are one of the major non-parenchymal cells in the liver and play various roles [19]. In the quiescent phase, HSCs store vitamin A in their cytoplasm [8]. Once activated by various stimuli, including cytokines (TGF-β), LPS and palmitic acid, HSCs promote liver fibrosis by producing extracellular matrix and inflammatory cytokines [8–10]. Moreover activated HSCs surrounding HCC tune the tumor microenvironment by secreting inflammatory cytokines, promoting HCC progression [18, 19]. In this study, we found that: 1) HSC activation by LPS and PA is regulated by PKR, and 2) IL-1β from activated HSCs contributes to HCC progression.

In the case of NASH, there is increased penetration of LPS and absorption of palmitic acid through the intestinal wall in addition to increased influx of LPS and palmitic acid into the liver via the portal vein [14, 15, 27]. Previous studies also showed that serum concentrations of LPS and palmitic acid were associated with the severity of both NASH and HCC [27, 28]. In addition to direct damage to hepatocytes, LPS and palmitic acid stimulate various kinds of immune cells to secrete various inflammatory cytokines, including IL-1β, inducing inflammation [29, 30]. LPS and palmitic acid activate HSCs and promote inflammatory cytokine production [16, 20, 31]. In our study, stimulation of HSCs by LPS and palmitic acid promoted IL-1β production from HSCs. Previous studies showed that IL-1β enhances tumorigenesis and the invasiveness of malignant tumors, including HCC, because IL-1β is a key cytokine for maintaining tumor microenvironment [32–35].

In this study, we found that IL-1β from activated HSCs promoted the proliferation and invasiveness of hepatoma cells. The amount of IL-1β secreted by HSCs might be less than that secreted by monocytes and dendritic cells [36, 37]. However, HSCs are known to be a major component of the liver, constituting almost one-third of the non-parenchymal cells in the liver, and are highly involved in the formation of the tumor microenvironment in HCCs [8]. Thus, we believe that IL-1β from HSCs is an important factor in the development of HCC [18].

**Fig 5:**
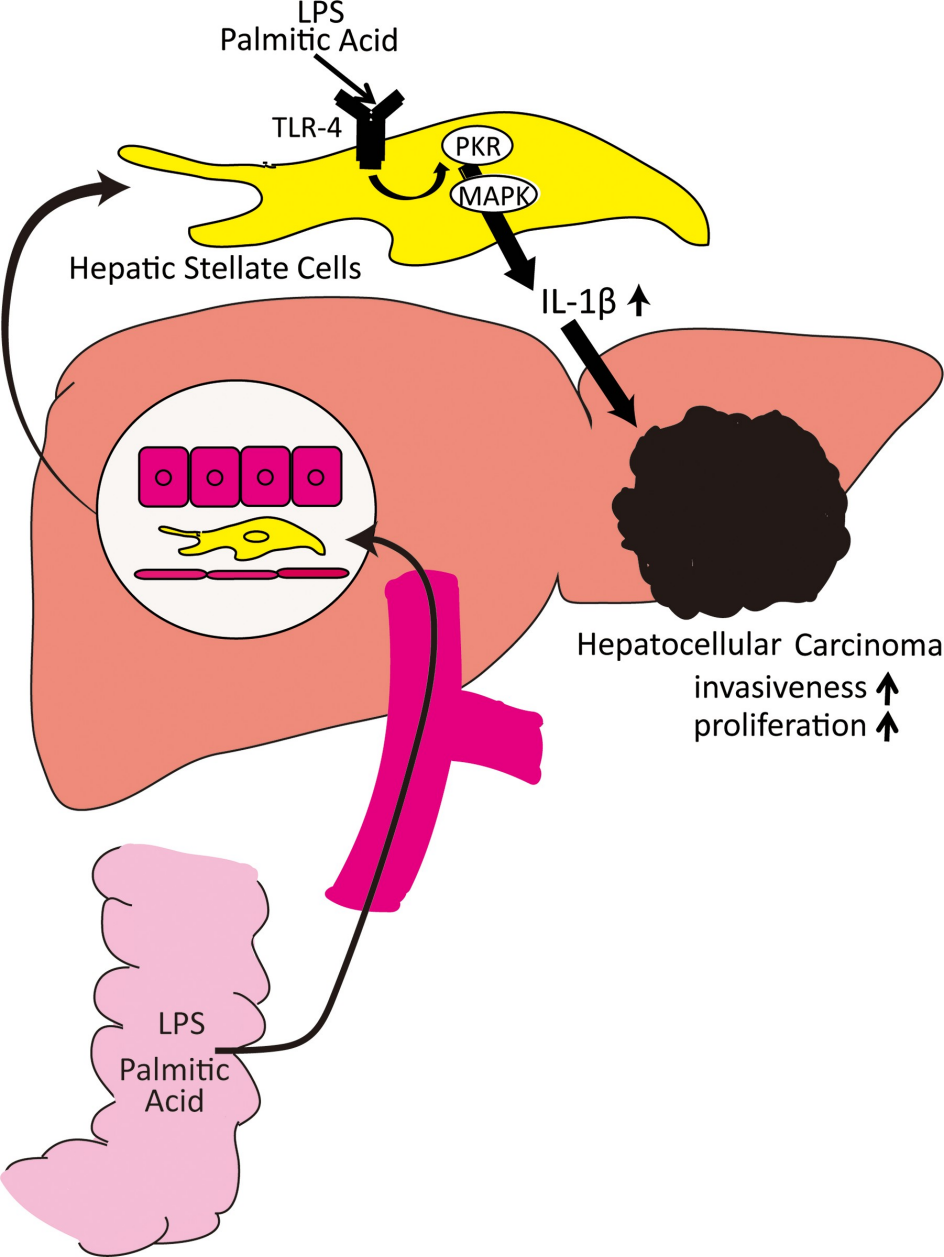
Proposed model of interplay between hepatic stellate cells and hepatocellular carcinoma. In nonalcoholic steatohepatitis cases, LPS and palmitic acid from the intestine stimulate HSCs and upregulate PKR expression. Upregulation of PKR increases IL-1β production via MAPK pathways. The secreted IL-1β enhances the progression of hepatocellular carcinoma.

To date, detailed mechanisms of IL-1β production by HSCs are not well understood. We hypothesized that PKR regulates IL-1β production in HSCs, because we have extensively studied PKR in hepatocytes and have found it to play a critical role in the immune response. We previously showed that PKR inhibits HCV infection by suppressing replication of HCV-infected hepatocytes [21]. In addition, Hiasa et al. reported that PKR was highly expressed in HCC tissues in the hepatitis C virus-infected liver and was involved in HCC development [22, 23]. Other groups reported that PKR in macrophages is involved in cytokine production via the MAPK pathway [25, 26]. Another group also suggested that PKR was involved in IL-1β production in HSCs [38]. In our study, we confirmed IL-1β production in HSCs, and demonstrated that IL-1β production was mediated by the MAPK pathway and was enhanced by phosphorylation of PKR (Fig 5). Further, we showed that PKR inhibition in HSCs reduced IL-1β production from HSCs and suppressed both proliferation and invasiveness of HCC.

In conclusion, PKR in HSCs promotes IL-1β production after stimulation, which enhances HCC development. PKR inhibition in HSCs suppresses the development of HCC by altering the cancer microenvironment. Therefore, PKR could be a therapeutic target for suppressing HCC, especially in cases of NASH, in which LPS and PA are involved in its pathogenesis.

## Supporting information

S1 Data The raw data for result of Fig 3B. (TIF)

S2 Data The raw data for result of Fig 4E. (TIF)
