# Supplementary figures and images for: Stimulated hepatic stellate cell promotes progression of hepatocellular carcinoma due to protein kinase R activation
Source: PLoS One. 2019 Feb 22;14(2):e0212589. doi: 10.1371/journal.pone.0212589 (PMC6386440; doi:10.1371/journal.pone.0212589)

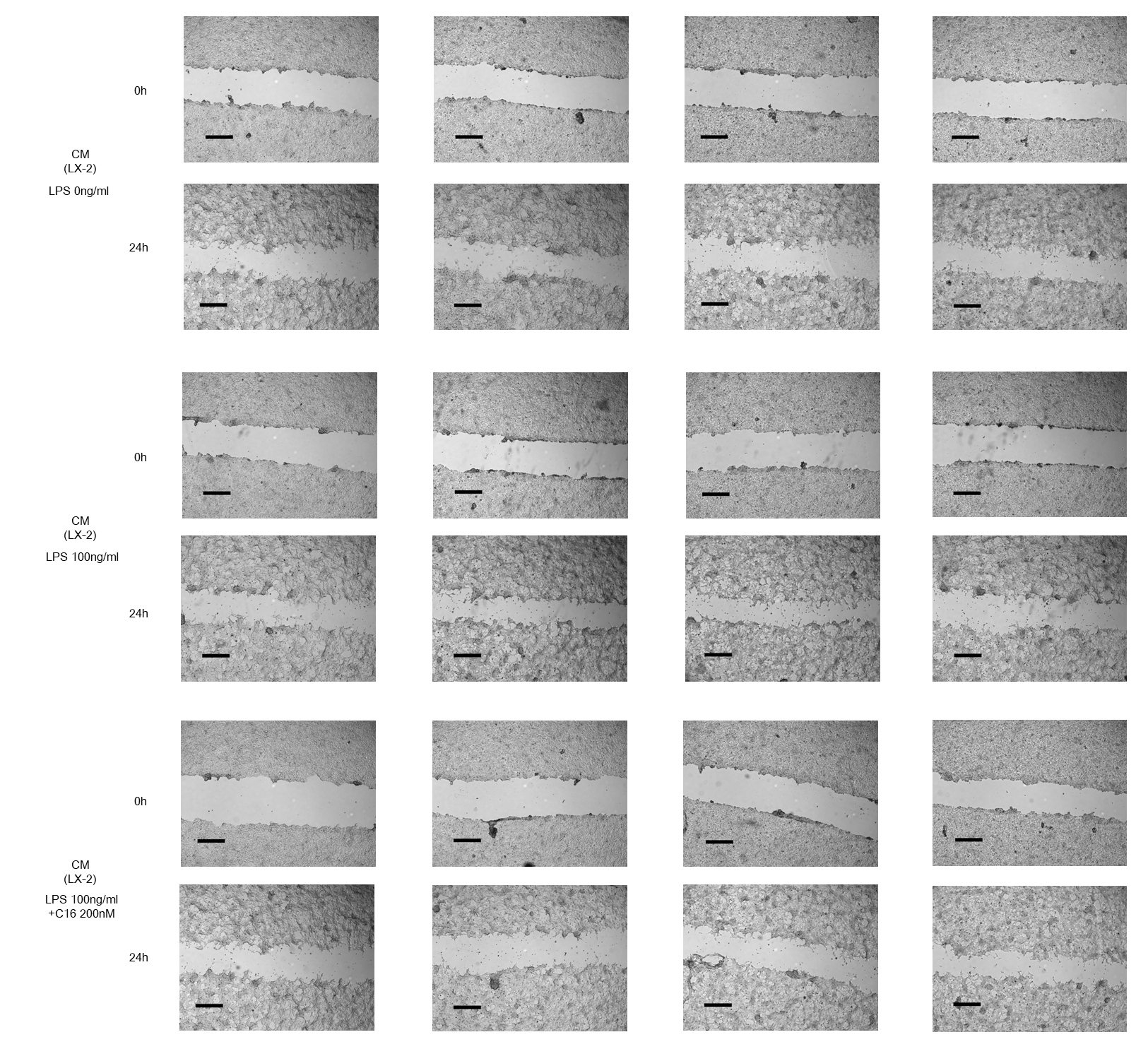

Supplement: S1 Data — (TIF) [file pone.0212589.s001.tif]

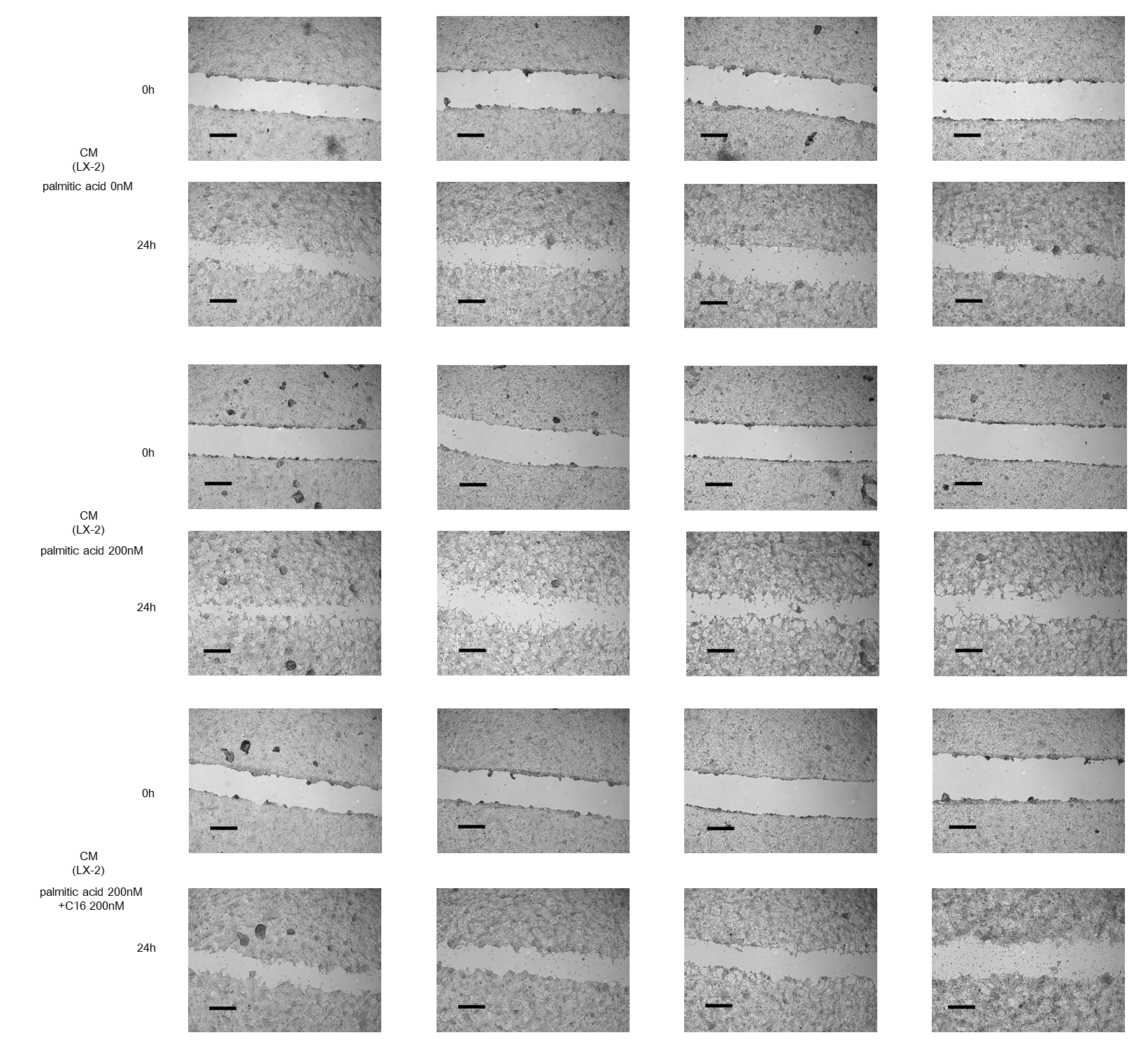

Supplement: S2 Data — (TIF) [file pone.0212589.s002.tif]
